# Social Determinants of Health and Health-Related Social Needs Among Adults With Chronic Diseases in the United States, Behavioral Risk Factor Surveillance System, 2022

**DOI:** 10.5888/pcd21.240362

**Published:** 2024-11-27

**Authors:** Karen Hacker, Craig W. Thomas, Guixiang Zhao, J’Neka S. Claxton, Paul Eke, Machell Town

**Affiliations:** 1 National Center for Chronic Disease Prevention and Health Promotion, Centers for Disease Control and Prevention, Atlanta, Georgia; 2 National Center for Chronic Disease Prevention and Health Promotion, Division of Population Health, Centers for Disease Control and Prevention, Atlanta, Georgia

## Abstract

**Introduction:**

The relationship between social determinants of health (SDOH) and health-related social needs (HRSN) and some chronic diseases at the population level is not well known. We sought to determine relationships between SDOH/HRSN and major chronic diseases among US adults by using data from the 2022 Behavioral Risk Factor Surveillance System (BRFSS).

**Methods:**

We used data from the new Social Determinants and Health Equity (SD/HE) module, conducted in 39 states, the District of Columbia, and 2 territories as part of the 2022 BRFSS. These data yielded a sample of 324,631 adult participants (aged ≥18 y). We examined 12 indicators of SDOH/HRSN and 9 chronic diseases. We calculated weighted prevalence estimates for each SDOH/HRSN measure for each chronic disease and associations between each SDOH/HRSN and each chronic disease.

**Results:**

Two-thirds of participants (66.3%) had 1 or more chronic diseases, and 59.4% reported 1 or more adverse SDOH/HRSN. Prevalence estimates for individual SDOH/HRSN measures were generally higher among participants with chronic diseases (except cancer). The more chronic diseases reported, the more likely participants were to have SDOH/HRSN (*P* < .05 for linear trend). The leading SDOH/HRSN measures associated with each chronic disease varied; however, the most common were mental stress, receiving food stamps or participating in the Supplemental Nutrition Assistance Program, cost as a barrier for needed medical care, and life dissatisfaction.

**Conclusion:**

From a treatment and prevention perspective, health care providers should consider the influence of SDOH/HRSN on people with or at risk for chronic diseases. Additionally, human service and public health systems in communities with high rates of chronic disease should consider these findings as they plan to mitigate adverse SDOH.

**Summary**

**What is already known on this topic?**

Health-related social needs (HRSN) experienced at the individual level may exacerbate health conditions. Early research suggests a relationship between some chronic diseases and some social needs.

**What is added by this report?**

We found that the more chronic conditions people had, the more social needs they identified. The leading social determinants of health (SDOH)/HRSN measures associated with each chronic disease varied; however, the most common were cost as a barrier for needed medical care, mental stress, and receiving food stamps or participating in the Supplemental Nutrition Assistance Program.

**What are the implications for public health practice?**

Having data on HRSN and SDOH can provide information for public health decision-making and resource allocation, especially during emergencies, when people with chronic conditions may be particularly vulnerable.

## Introduction

Chronic conditions such as diabetes, heart disease, and cancer are among the leading causes of death and disability in the US. They account for more than $1 trillion in health care costs annually (1,2). The leading risk factors for chronic disease are tobacco use, obesity and poor nutrition, physical inactivity, and excessive alcohol use (2). However, the nonmedical factors that influence health, often referred to as social determinants of health (SDOH) (3), drive as much as 50% of health outcomes and support or deter individual behavior (4–6). SDOH surround us, whether related to food, housing, transportation, safety and security, or discriminatory policies that influence individual health-related experiences (7,8). SDOH also contribute to health disparities, which became especially apparent during the COVID-19 pandemic (9). Access to health care, employment, and housing were related to contracting COVID-19 (9), and chronic disease (diabetes, cancer, obesity, and heart disease) proved to be a risk factor for severe COVID-19 (10). Linking this type of information was helpful to communities as they made decisions about COVID-19 testing and vaccination (11,12).

Health-related social needs (HRSN) are experienced at the individual level (eg, food insecurity, housing instability) and may exacerbate health conditions (13). A recent rule from the Centers for Medicare & Medicaid Services requires health care providers to screen for HRSN, and Medicaid and Medicare reimbursement opportunities are available (14,15). Referring patients to HRSN-related services and addressing HRSN may help reduce health care costs (16). Emerging research also suggests a strong relationship between chronic diseases and HRSN (17,18). Information on SDOH at the population level could also be helpful for public health decision-making (19). However, both SDOH and HRSN data at the population level are generally lacking, and health care system data can be challenging to obtain for public health departments and community agencies (20,21).

To address the gap in population-based data on SDOH/HRSN, the Centers for Disease Control and Prevention (CDC) introduced a new Social Determinants and Health Equity (SD/HE) module in the 2022 Behavioral Risk Factor Surveillance System (BRFSS). The new module includes items that assess various SDOH and HRSN, including housing instability, transportation, and food security (22). Although the BRFSS collects SDOH/HRSN information from individuals, the data are analyzed and reported at the population level by using statistical methods to derive state-based prevalence estimates of SDOH/HRSN. The objective of this study was to describe the relationship between self-reported SDOH/HRSN and chronic disease using data from the 2022 BRFSS SD/HE module.

## Methods

### Data source and study population

The BRFSS is a state-based landline and cellular telephone survey that collects data from noninstitutionalized US civilian residents aged 18 years or older on their health-related risk behaviors, chronic illnesses and conditions, health care access, and use of health-related services (23). It is conducted annually in all 50 states, the District of Columbia, and participating US territories. The dataset is public and deidentified. The BRFSS is composed of standard questions in the core section asked of all states and territories and additional sets of questions on particular topics in the optional modules asked of some states and territories. Detailed information on the BRFSS survey design, sampling, data collection, and weights are described elsewhere (23). For the 2022 BRFSS, the median response rate was 45.1%, ranging from 22.8% in Guam to 66.8% in South Dakota. Despite relatively low response rates, BRFSS results are comparable to results from other national surveys (24,25).

### SDOH and HRSN measures

In 2022, the BRFSS introduced a new SD/HE optional module with 10 questions; data were collected from 39 states, the District of Columbia, and 2 US territories (Puerto Rico and US Virgin Islands). The 11 states that did not use the SD/HE module were Arkansas, Colorado, Hawaii, Illinois, Louisiana, Maryland, Michigan, Nebraska, New York, North Dakota, and South Dakota. The SDOH/HRSN measures used in the SD/HE module are based on the Centers for Medicare & Medicaid Services Center for Medicare and Medicaid Innovation’s HRSN Screening Tool (26), and they cover the following 10 topics: life satisfaction, social and emotional support, social isolation or loneliness, employment stability, receipt of food stamps or SNAP (Supplemental Nutrition Assistance Program), food security, housing security, stability of utility services (eg, gas, water), transportation access, and mental well-being. Details on the module are available elsewhere (22,27). Two additional SDOH measures were from the BRFSS Health Care Access Core Section (health insurance coverage and cost as a barrier for needed medical care). This study examined 12 adverse SDOH/HRSN (Table 1). An SDOH/HRSN summary score was calculated for participants who had a valid response for the SDOH/HRSN questions. Each SDOH/HRSN was scored as 1, so the total score ranged from 0 to 12 for each respondent. The number of SDOH/HRSN was categorized as 0, 1, 2, 3, 4, and ≥5.

**Table 1:** Summary and Definitions of SDOH/HRSN Measures With Corresponding Question Asked and Scores Assigned, 39 States, the District of Columbia, and 2 US Territories, Behavioral Risk Factor Surveillance System, 2022

Adverse SDOH/HRSN	Question asked	Answer options	
		Score = 1	Score = 0
Life dissatisfaction	In general, how satisfied are you with your life? Are you . . .	Dissatisfied/very dissatisfied	Very satisfied/satisfied
Lack of social and emotional support	How often do you get the social and emotional support that you need? Is that . . .	Sometimes/rarely/never	Always/usually
Socially isolated/loneliness	How often do you feel socially isolated from others? Is it . . .	Always/usually/sometimes	Never/rarely
Loss or reduced hours for employment	In the past 12 months have you lost employment or had hours reduced?	Yes	No
Receiving food stamps/SNAP	During the past 12 months, have you received food stamps, also called SNAP, the Supplemental Nutrition Assistance Program on an EBT card?	Yes	No
Food insecurity	During the past 12 months how often did the food that you bought not last, and you didn’t have money to get more? Was that . . .	Always/usually/sometimes	Never/rarely
Housing insecurity	During the last 12 months, was there a time when you were not able to pay your mortgage, rent or utility bills?	Yes	No
Threat to shut off utility services	During the last 12 months was there a time when an electric, gas, oil, or water company threatened to shut off services?	Yes	No
Lack of reliable transportation	During the past 12 months has a lack of reliable transportation kept you from medical appointments, meetings, work, or from getting things needed for daily living?	Yes	No
Mental stress	Stress means a situation in which a person feels tense, restless, nervous or anxious, or is unable to sleep at night because their mind is troubled all the time. Within the last 30 days, how often have you felt this kind of stress? Was it . . .	Always/usually	Sometimes/rarely/never
Lack of health insurance	What is the current primary source of your health insurance?	No coverage of any type	Having any type of insurance
Cost barrier for needed medical care	Was there a time in the past 12 months when you needed to see a doctor but could not because you could not afford it?	Yes	No

Abbreviations: EBT, electronic benefits transfer; HRSN: health-related social needs; SDOH: social determinants of health; SNAP, Supplemental Nutrition Assistance Program.

### Health outcomes

We assessed the following 9 chronic diseases: arthritis, cancer, chronic respiratory disease, coronary heart disease (CHD), current asthma, depression, diabetes, obesity, and stroke. Arthritis was defined as participants reporting they had ever been told they had some form of arthritis, rheumatoid arthritis, gout, lupus, or fibromyalgia. Cancer was defined as participants reporting they had ever been told they had any type of cancer except those reporting only nonmelanoma skin cancer. Chronic respiratory disease was defined as participants reporting they had ever been told they had chronic obstructive pulmonary disease (COPD), emphysema, or chronic bronchitis. CHD was defined as participants reporting they had ever been told they had either a heart attack/myocardial infarction or angina/coronary heart disease. Current asthma was defined as participants reporting they had ever been told they had asthma and still had asthma at the time they responded. Depression was defined as participants reporting they had ever been told they had a depressive disorder (including depression, major depression, dysthymia, or minor depression). Diabetes was defined as participants reporting they had ever been told they had diabetes; we excluded gestational diabetes and prediabetes. Obesity was defined as participants having a body mass index (BMI) of 30.0 or more, calculated by using self-reported weight (in kilograms) divided by the square of self-reported height (in meters). Stroke was defined as participants reporting they had ever been told they had a stroke. For the 9 chronic diseases, we grouped participants into 6 categories: having 0, 1, 2, 3, 4 or 5 or more chronic diseases. In addition, for the 5 preventable chronic diseases that are among the leading causes of death in the US (ie, cancer, CHD, chronic respiratory diseases, diabetes, and stroke,) we grouped participants into 4 categories: having 0, 1, 2, or 3 or more of these diseases.

### Study covariates

Study covariates were demographic variables and chronic disease risk factors. Demographic characteristics were age (18–34, 35–49, 50–64, and ≥65 y), sex (male and female), race and ethnicity (Hispanic, non-Hispanic American Indian or Alaska Native, non-Hispanic Asian, non-Hispanic Black or African American, non-Hispanic Native Hawaiian or Other Pacific Islander, non-Hispanic White, and non-Hispanic multiracial), education (less than high school diploma, high school graduate/GED [General Educational Development], some college/technical school, 4 years of college or more), marital status (married, previously married [divorced, widowed, or separated], and never married or living with a partner), and annual household income (<$25,000, $25,000 to <50,000, $50,000 to <75,000, $75,000 to <100,000, $100,000 to <150,000, ≥$150,000, or unknown). Physical inactivity, current smoking, obesity, and alcohol use were used as risk factors for all diseases except obesity. Diabetes was also included as a risk factor for heart disease and stroke.

### Statistical analysis

Of 338,778 participants from the 39 states, the District of Columbia, and the 2 US territories that administered the 2022 BRFSS SD/HE optional module, 324,631 remained after excluding those who responded “don’t know/not sure,” refused to answer, or had missing responses for demographic variables (except for those with missing values for income). Participants with missing information on a chronic disease (eg, stroke) or an SDOH/HRSN measure (eg, loneliness) were further excluded from the analysis on that disease (stroke) or that SDOH/HRSN measure (loneliness). Missing data for the 9 chronic diseases ranged from 0.2% for diabetes to 10.3% for obesity. Missing data for the 12 SDOH/HRSN measures ranged from 0.3% for cost as a barrier for needed medical care to 17.6% for mental stress. For calculating the number of participants who reported having 0, 1 ,2 ,3, or 4 chronic diseases or SDOH/HRSN, we included all participants who had a valid answer for each of the 9 chronic diseases or 12 SDOH/HRSN questions. For calculating the number of participants who reported having 5 or more chronic diseases or SDOH/HRSN, we included participants who had a valid answer for at least 5 questions. We found 281,667 participants who had valid data for calculating the number of the 9 chronic diseases and 250,550 participants who had valid data for calculating the number of adverse SDOH/HRSN measures. We calculated weighted prevalence estimates with 95% CIs for individual adverse SDOH/HRSN measures and the number of adverse SDOH/HRSN overall, by individual chronic diseases, and by the total number of chronic diseases reported. Significance was determined according to whether the 95% CIs for any 2 estimates overlapped.

We assessed associations between SDOH/HRSN and chronic diseases by conducting log-linear regression analyses and estimating adjusted prevalence ratios (APRs) with 95% CIs after adjusting for demographic variables and disease-specific risk factors. To assess the strength of the associations of individual adverse SDOH/HRSN with each chronic disease, we conducted a full model fit by including all SDOH/HRSN measures simultaneously to account for correlations, with adjustment for demographic variables and disease-specific risk factors. We tested multicollinearity among SDOH/HRSN measures by estimated correlation matrix and variance inflation factors (VIFs) and detected no strong collinearity (correlation coefficients were <0.5 for all and VIFs were <1.7 for all). We used SAS-callable SUDAAN software release 11.0.3 (Research Triangle Institute) for analysis to account for the multistage, complex sampling design. These methods are described elsewhere (17,28). This activity was reviewed by CDC and conducted consistent with applicable federal law and CDC policy.

## Results

In the population that participated in the 2022 BRFSS SD/HE module, 29.3% were aged 18 to 34 years; age groups of 35 to 49, 50 to 64, and 65 years or older were almost equally distributed (Table 2). Men accounted for 48.4% of participants. By race and ethnicity, the largest proportions were non-Hispanic White (57.4%), non-Hispanic Black or African American (11.6%), and Hispanic (20.2%). Twelve percent had less than a high school diploma, and 29.8% had 4 years of college or more. Approximately half (50.1%) were married. Approximately one-third (33.0%) had an annual household income below $50,000; one-fifth had an annual household income of $50,000 to less than $100,000 (22.1%) or $100,000 or more (22.0%). Just over one-fifth (23.1%) provided no information on income. Approximately two-thirds (66.3%) had 1 or more chronic diseases; 59.4% reported 1 or more adverse SDOH/HRSN, and 11.1% reported 5 or more SDOH/HRSN.

**Table 2:** Demographic Distribution of Study Population, 39 States, the District of Columbia, and 2 US Territories, Behavioral Risk Factor Surveillance System, 2022

Characteristic	No.	Weighted %^a^ (95% CI)
**Overall**	324,631^b^	100.0
**Age, y**		
18–34	53,765	29.3 (28.9–29.6)
35–49	64,167	23.4 (23.1–23.7)
50–64	87,289	24.5 (24.2–24.8)
≥65	119,410	22.9 (22.6–23.2)
**Sex**		
Male	151,464	48.4 (48.0–48.8)
Female	173,167	51.6 (51.2–52.0)
**Race and ethnicity**		
Hispanic	33,552	20.2 (19.9–20.6)
Non-Hispanic American Indian or Alaska Native	4,765	1.3 (1.2–1.4)
Non-Hispanic Asian	7,566	6.0 (5.8–6.3)
Non-Hispanic Black or African American	25,941	11.6 (11.4–11.9)
Non-Hispanic Native Hawaiian or Other Pacific Islander	692	0.4 (0.4–0.5)
Non-Hispanic White	246,091	57.4 (57.0–57.7)
Non-Hispanic multiracial	6,024	3.1 (2.9–3.2)
**Education**		
Less than high school diploma	19,063	12.0 (11.6–12.3)
High school graduate/GED	79,545	27.5 (27.1–27.8)
Some college	88,730	30.7 (30.4–31.1)
4 Years of college or more	137,293	29.8 (29.5–30.2)
**Marital status**		
Married	169,024	50.1 (49.7–50.5)
Previously married	84,568	20.0 (19.7–20.2)
Never married or living with a partner	71,039	30.0 (29.6–30.3)
**Annual household income, $**		
<25,000	40,950	13.2 (12.9–13.5)
25,000 to <50,000	65,486	19.8 (19.5–20.0)
50,000 to <75,000	43,740	12.0 (11.8–12.2)
75,000 to <100,000	35,845	10.1 (9.9–10.3)
100,000 to <150,000	37,442	10.9 (10.7–11.1)
≥150,000	33,940	11.1 (10.8–11.3)
Unknown	67,228	23.1 (22.7–23.4)
**No. of chronic diseases (n = 281,667)^c^**		
0	80,673	33.7 (33.3–34.1)
1	85,178	31.0 (30.6–31.4)
2	57,435	18.3 (18.0–18.6)
3	32,076	9.5 (9.3–9.7)
4	15,167	4.2 (4.1–4.4)
≥5	11,138	3.2 (3.1–3.4)
**No. of adverse SDOH/HRSN (n = 250,550)^d^**		
0	118,955	40.6 (40.2–41.0)
1	55,271	22.3 (22.0–22.7)
2	29,447	12.9 (12.7–13.2)
3	16,288	7.9 (7.6–8.1)
4	10,383	5.2 (5.0–5.4)
≥5	20,206	11.1 (10.8–11.4)

Abbreviations: GED, General Educational Development; HRSN, health-related social needs; SDOH, social determinants of health.

a Percentages may not add to 100 because of rounding.

b Includes participants with valid demographic information (participants with missing information on income were included for analysis).

c Includes participants who had valid information for computing the number of chronic diseases. The following chronic diseases were counted: arthritis, cancer, chronic respiratory diseases, coronary heart disease, current asthma, depression, diabetes, obesity, and stroke.

d Includes participants who had valid information for computing the number of adverse SDOH and HRSN.

Among all study participants, social isolation/loneliness and lack of social and emotional support were the most commonly reported SDOH/HRSN measures, accounting for 31.9% and 24.8%, respectively. Most prevalence estimates for individual SDOH/HRSN were higher among participants with chronic diseases than among those without (Table 3). However, we found a few exceptions. First, the prevalence of lack of health insurance was significantly lower among participants with reported chronic diseases than among those without. Second, the prevalence of the adverse SDOH/HRSN (except for life dissatisfaction and lack of reliable transportation) were significantly lower among adults with cancer than those without cancer.

**Table 3:** Weighted Percentages of Having Individual Adverse SDOH/HRSN Among Adults with Chronic Diseases, 39 States, the District of Columbia, and 2 US Territories, Behavioral Risk Factor Surveillance System, 2022^a^

Chronic disease	Life dissatisfaction	Lack of social or emotional support	Social isolation or loneliness	Lost or reduced hours for employment	Receiving food stamps or SNAP	Food insecure	Housing insecure	Threat to shut off utility services	Lack of reliable transportation	Mental stress	Lack of health insurance	Cost barrier for needed medical care
Sample size, n	269,677	268,104	269,206	269,190	270,024	269,124	269,041	269,061	268,828	267,435	312,715	323,646
**Overall**	6.2 (6.0–6.5)	24.8 (24.4–25.2)	31.9 (31.5–32.3)	12.7 (12.4–13.0)	12.3 (12.1–12.6)	14.0 (13.7–14.3)	11.9 (11.6–12.2)	7.5 (7.3–7.8)	8.1 (7.9–8.4)	14.2 (13.9–14.5)	8.8 (8.6–9.1)	11.4 (11.1–11.6)
**Arthritis**												
Yes	8.0 (7.6–8.4)	25.3 (24.7–26.0)	33.5 (32.8–34.2)	9.4 (8.9–9.8)	14.9 (14.4–15.5)	16.0 (15.5–16.6)	12.7 (12.2–13.2)	8.9 (8.4–9.3)	9.5 (9.0–9.9)	16.3 (15.7–16.8)	4.0 (3.7–4.3)	11.3 (10.9–11.8)
No	5.5 (5.2–5.7)	24.5 (24.0–24.9)	31.2 (30.7–31.6)	13.9 (13.5–14.3)	11.3 (11.0–11.6)	13.1 (12.8–13.5)	11.5 (11.1–11.8)	7.0 (6.7–7.2)	7.6 (7.3–7.8)	13.3 (12.9–13.7)	10.6 (10.3–10.9)	11.3 (11.0–11.6)
**Cancer^b^**												
Yes	7.1 (6.3–8.0)	21.0 (20.0–22.1)	29.1 (28.0–30.3)	7.9 (7.0–8.8)	10.2 (9.4–11.1)	12.0 (11.0–13.0)	8.9 (8.1–9.9)	6.4 (5.7–7.1)	7.5 (6.6–8.4)	12.7 (11.7–13.7)	2.7 (2.2–3.2)	8.9 (8.0–9.8)
No	6.1 (5.9–6.3)	25.0 (24.6–25.4)	32.0 (31.6–32.5)	13.1 (12.8–13.4)	12.5 (12.2–12.8)	14.1 (13.8–14.4)	12.1 (11.8–12.4)	7.6 (7.4–7.9)	8.2 (7.9–8.4)	14.3 (14.0–14.6)	9.4 (9.1–9.6)	11.5 (11.3–11.8)
**Chronic respiratory disease**												
Yes	12.2 (11.3–13.1)	33.8 (32.5–35.2)	42.1 (40.8–43.5)	11.8 (10.7–12.9)	23.8 (22.6–25.0)	26.3 (25.1–27.6)	19.8 (18.6–21.0)	14.6 (13.5–15.7)	16.8 (15.7–17.9)	22.8 (21.7–24.0)	5.4 (4.9–6.0)	17.4 (16.3–18.6)
No	5.7 (5.5–6.0)	24.0 (23.6–24.4)	31.0 (30.6–31.4)	12.7 (12.4–13.0)	11.4 (11.1–11.7)	13.0 (12.7–13.3)	11.2 (10.9–11.5)	7.0 (6.7–7.2)	7.5 (7.2–7.7)	13.5 (13.1–13.8)	9.1 (8.8–9.3)	10.9 (10.6–11.1)
**Coronary heart disease**												
Yes	9.5 (8.7–10.4)	28.5 (27.2–29.8)	34.0 (32.8–35.3)	9.0 (8.1–10.0)	17.9 (16.8–19.0)	20.0 (18.8–21.3)	14.3 (13.3–15.4)	10.1 (9.2–11.1)	12.4 (11.3–13.4)	16.6 (15.5–17.8)	3.9 (3.4–4.5)	13.3 (12.3–14.4)
No	5.9 (5.7–6.1)	24.3 (23.9–24.7)	31.5 (31.1–32.0)	12.9 (12.6–13.3)	11.8 (11.5–12.1)	13.4 (13.0–13.7)	11.6 (11.3–11.9)	7.3 (7.0–7.5)	7.7 (7.5–8.0)	13.9 (13.6–14.2)	9.2 (8.9–9.4)	11.1 (10.9–11.4)
**Current asthma**												
Yes	11.2 (10.4–12.1)	29.7 (28.6–30.9)	42.3 (41.1–43.5)	15.5 (14.5–16.5)	22.1 (21.0–23.2)	23.3 (22.2–24.4)	20.7 (19.6–21.9)	13.9 (13.0–14.9)	15.4 (14.4–16.5)	25.1 (24.0–26.2)	6.3 (5.7–7.0)	17.3 (16.4–18.3)
No	5.6 (5.4–5.8)	24.0 (23.6–24.4)	30.5 (30.1–30.9)	12.3 (12.0–12.6)	11.2 (10.9–11.5)	12.9 (12.6–13.2)	10.8 (10.5–11.1)	6.8 (6.5–7.0)	7.2 (7.0–7.5)	12.8 (12.5–13.1)	9.1 (8.8–9.3)	10.6 (10.3–10.8)
**Depression**												
Yes	16.5 (15.9–17.2)	38.3 (37.5–39.1)	55.8 (55.0–56.6)	18.1 (17.4–18.8)	20.5 (19.8–21.2)	22.9 (22.2–23.7)	21.2 (20.5–21.9)	13.8 (13.2–14.4)	16.5 (15.9–17.2)	35.7 (34.9–36.5)	7.7 (7.2–8.1)	20.2 (19.6–20.9)
No	3.3 (3.1–3.5)	20.9 (20.4–21.3)	25.1 (24.7–25.5)	11.1 (10.8–11.5)	10.1 (9.8–10.4)	11.5 (11.1–11.8)	9.3 (9.0–9.6)	5.8 (5.6–6.0)	5.8 (5.5–6.0)	8.2 (7.9–8.4)	9.1 (8.8–9.4)	9.0 (8.7–9.2)
**Diabetes**												
Yes	7.9 (7.3–8.5)	27.6 (26.6–28.7)	32.1 (31.1–33.2)	9.9 (9.1–10.7)	17.7 (16.8–18.7)	19.7 (18.7–20.7)	14.1 (13.2–15.0)	9.5 (8.8–10.3)	10.3 (9.5–11.1)	13.5 (12.8–14.3)	5.0 (4.5–5.6)	11.6 (10.8–12.4)
No	6.0 (5.8–6.2)	24.3 (23.9–24.7)	31.8 (31.4–32.2)	13.1 (12.7–13.4)	11.5 (11.2–11.8)	13.1 (12.8–13.5)	11.5 (11.2–11.8)	7.2 (7.0–7.5)	7.8 (7.5–8.0)	14.3 (13.9–14.6)	9.3 (9.1–9.6)	11.3 (11.0–11.6)
**Obesity**												
Yes	7.1 (6.8–7.5)	25.7 (25.1–26.4)	32.9 (32.2–33.5)	13.2 (12.7–13.8)	15.8 (15.3–16.4)	16.3 (15.8–16.9)	14.4 (13.9–15.0)	9.5 (9.1–9.9)	8.7 (8.3–9.2)	16.1 (15.6–16.6)	8.0 (7.5–8.4)	12.6 (12.2–13.1)
No	5.8 (5.6–6.1)	24.4 (23.9–24.9)	31.4 (30.8–31.8)	12.4 (12.0–12.8)	10.4 (10.0–10.7)	12.2 (11.8–12.6)	10.3 (10.0–10.7)	6.5 (6.2–6.8)	7.8 (7.4–8.1)	13.5 (13.1–13.9)	8.4 (8.1–8.7)	10.5 (10.2–10.8)
**Stroke**												
Yes	11.3 (10.0–12.7)	31.1 (29.1–33.0)	37.6 (35.6–39.6)	10.8 (9.4–12.4)	22.4 (20.7–24.2)	26.0 (24.0–28.1)	18.2 (16.6–20.0)	12.5 (11.1–14.1)	16.2 (14.6–17.9)	18.8 (17.2–20.6)	4.6 (3.9–5.5)	15.3 (13.7–17.1)
No	6.0 (5.8–6.2)	24.5 (24.1–24.8)	31.6 (31.2–32.0)	12.7 (12.4–13.0)	11.9 (11.6–12.2)	13.5 (13.2–13.8)	11.6 (11.3–11.9)	7.3 (7.1–7.5)	7.8 (7.6–8.0)	14.0 (13.6–14.3)	9 (8.7–9.2)	11.2 (10.9–11.4)

Abbreviations: HRSN, health-related social needs; SDOH, social determinants of health; SNAP, Supplemental Nutrition Program.

a Sample sizes reported for individual SDOH and HRSN measures varied due to missing data for each measure (ranging from 0.3% for cost barrier for needed medical care to 17.6% for mental stress); sample sizes were further reduced after excluding participants with missing on chronic disease (ranging from 0.2% for diabetes to 10.3% for obesity). Data are % (95% CI).

b Includes all cancers except nonmelanoma skin cancers.

When we examined data on individual participants with various chronic diseases, between 50.4% (cancer) and 80.5% (depression) had 1 or more SDOH/HRSN (Table 4). In general, the percentage of participants reporting multiple adverse SDOH/HRSN was significantly higher among those with chronic diseases than those without. Specifically, the percentages of participants having 5 or more adverse SDOH/HRSN were significantly higher among participants with each chronic disease than those without except for cancer. The percentages of participants having 4 to 5 or more adverse SDOH/HRSN were significantly higher among adults with arthritis, diabetes, and stroke than among adults without these conditions. The percentages of adults having 3 to 5 or more adverse SDOH/HRSN were significantly higher among adults with current asthma than among adults without current asthma. The percentages of adults having 2 to 5 or more adverse SDOH/HRSN were significantly higher among those with chronic respiratory diseases, depression, and obesity than among those without these conditions. In contrast, the percentages of adults having 2 to 5 or more adverse SDOH/HRSN were significantly lower among adults with cancer than among adults without cancer.

**Table 4:** Weighted Percentages of Having a Chronic Disease and Number of Adverse SDOH and HRSN Among Adults (N = 250,550) With Valid Responses to SDOH/HRSN Measures, Behavioral Risk Factor Surveillance System, 2022

Chronic disease	No. (weighted %)	No. of adverse SDOH/HRSN, % (95% CI)					
		0	1	2	3	4	≥5
**Overall**	250,550 (100)	40.6 (40.2–41.0)	22.3 (22.0–22.7)	12.9 (12.7–13.2)	7.9 (7.6–8.1)	5.2 (5.0–5.4)	11.1 (10.8–11.4)
**Arthritis**							
Yes	88,882 (28.3)	40.9 (40.2–41.6)	21.5 (20.9–22.1)	12.3 (11.8–12.7)	7.4 (7.0–7.7)	5.6 (5.3–6.0)	12.4 (11.9–12.9)
No	160,525 (71.7)	40.6 (40.1–41.0)	22.7 (22.2–23.1)	13.2 (12.9–13.6)	8.1 (7.8–8.4)	5.0 (4.8–5.2)	10.5 (10.1–10.9)
**Cancer^a^**							
Yes	30,091 (8.7)	49.6 (48.3–50.8)	21.8 (20.8–22.8)	10.7 (9.9–11.5)	5.7 (5.1–6.3)	3.5 (3.1–4.0)	8.7 (7.8–9.7)
No	219,343 (91.3)	39.8 (39.4–40.2)	22.4 (22.0–22.8)	13.2 (12.9–13.5)	8.1 (7.8–8.3)	5.4 (5.2–5.6)	11.2 (10.9–11.5)
**Chronic respiratory diseases**							
Yes	20,506 (7.1)	27.3 (26.1–28.6)	19.5 (18.3–20.7)	14.1 (13.1–15.1)	9.6 (8.9–10.4)	7.4 (6.8–8.1)	22.1 (20.8–23.4)
No	229,164 (92.9)	41.7 (41.3–42.1)	22.5 (22.1–22.9)	12.9 (12.6–13.2)	7.7 (7.5–8.0)	5.0 (4.8–5.2)	10.2 (9.9–10.5)
**Coronary heart disease**							
Yes	22,713 (7.2)	38.5 (37.2–39.8)	20.7 (19.6–21.8)	11.8 (11.0–12.7)	8.2 (7.4–9.1)	5.5 (4.9–6.1)	15.3 (14.2–16.5)
No	225,562 (92.8)	40.9 (40.5–41.3)	22.5 (22.1–22.9)	13.0 (12.7–13.3)	7.8 (7.6–8.1)	5.1 (4.9–5.3)	10.6 (10.3–10.9)
**Current asthma**							
Yes	26,644 (10.3)	29.1 (28.0–30.2)	19.7 (18.8–20.7)	13.2 (12.4–14.1)	9.4 (8.7–10.1)	7.3 (6.7–8.0)	21.3 (20.2–22.5)
No	221,980 (89.7)	42.1 (41.6–42.5)	22.6 (22.2–23.0)	12.9 (12.6–13.2)	7.6 (7.4–7.9)	4.9 (4.7–5.1)	9.8 (9.5–10.1)
**Depression**							
Yes	54,359 (21.9)	19.5 (18.9–20.2)	18.5 (17.8–19.1)	15.5 (14.9–16.1)	11.8 (11.3–12.4)	9.4 (8.9–9.9)	25.3 (24.5–26.1)
No	195,080 (78.1)	46.7 (46.2–47.1)	23.4 (23.0–23.9)	12.2 (11.9–12.6)	6.7 (6.4–7.0)	4.0 (3.8–4.2)	7.0 (6.7–7.3)
**Diabetes**							
Yes	35,042 (12.7)	36.9 (35.8–38.0)	22.6 (21.5–23.7)	13.9 (13.1–14.8)	8.1 (7.5–8.8)	5.5 (5.0–6.0)	13.0 (12.1–14.0)
No	215,145 (87.3)	41.2 (40.8–41.6)	22.3 (21.9–22.7)	12.8 (12.5–13.1)	7.8 (7.5–8.1)	5.2 (5.0–5.4)	10.8 (10.4–11.1)
**Obesity**							
Yes	81,307 (35.1)	37.3 (36.6–38.0)	21.8 (21.2–22.4)	13.7 (13.2–14.2)	8.5 (8.1–8.9)	6.0 (5.6–6.3)	12.7 (12.2–13.3)
No	154,774 (64.9)	42.6 (42.1–43.1)	22.7 (22.2–23.2)	12.5 (12.2–12.9)	7.5 (7.1–7.8)	4.6 (4.4–4.8)	10.1 (9.8–10.5)
**Stroke**							
Yes	10,667 (3.7)	31.5 (29.5–33.5)	20.7 (18.8–22.7)	13.2 (11.9–14.7)	7.9 (7.0–9.0)	6.9 (5.9–7.9)	19.8 (18.0–21.7)
No	239,254 (96.3)	41.0 (40.6–41.4)	22.4 (22.0–22.8)	12.9 (12.6–13.2)	7.8 (7.6–8.1)	5.2 (5.0–5.3)	10.7 (10.4–11.0)
**No. of chronic diseases that contribute to a high risk of mortality^b^**							
0	163,930 (72.3)	41.3 (40.8–41.8)	22.7 (22.3–23.2)	13.1 (12.8–13.5)	7.9 (7.6–8.2)	5.2 (4.9–5.4)	9.8 (9.5–10.2)
1	56,154 (19.3)	42.1 (41.2–43.0)	21.6 (20.8–22.4)	12.3 (11.7–12.9)	7.5 (7.0–8.1)	4.8 (4.4–5.1)	11.8 (11.1–12.5)
2	18,790 (6.1)	36.1 (34.7–37.5)	21.9 (20.6–23.3)	12.7 (11.7–13.7)	8.3 (7.4–9.3)	5.8 (5.2–6.6)	15.2 (14.0–16.5)
≥3	6,992 (2.3)	31.7 (29.3–34.1)	20.3 (18.4–22.3)	14.2 (12.7–16.0)	7.7 (6.8–8.7)	6.9 (5.6–7.9)	19.5 (17.2–22.0)

Abbreviations: HRSN, health-related social needs; SDOH, social determinants of health.

a Includes all cancers except nonmelanoma skin cancers.

b Includes coronary heart disease, cancer (but not nonmelanoma skin cancer), chronic respiratory diseases, diabetes, and stroke.

More than one-quarter (27.7%) of participants reported having 1 or more of the 5 preventable chronic diseases that are among the leading causes of death in the US (cancer, CHD, chronic respiratory diseases, diabetes, and stroke). The more chronic diseases reported, the more likely participants were to have HRSN. We found a significant linear increase in reporting 4 or 5 or more adverse SDOH/HRSN (*P* for linear trend <.05 for all, Table 4) for all conditions.

After adjustment for demographic characteristics and disease-specific risk factors, most individual SDOH/HRSN were associated with a higher prevalence of all chronic diseases. The only exception was lack of health insurance, which was significantly associated with a lower prevalence of all chronic diseases (data not shown). Overall, among the 12 SDOH/HRSN, mental stress, receiving food stamps or participating in SNAP, cost as a barrier for needed medical care, and life dissatisfaction were more frequently associated with chronic diseases (Figure). However, we found differences by chronic disease. Cost as a barrier for needed medical care was the strongest measure associated with CHD (APR = 1.44; 95% CI, 1.30–1.59) and chronic respiratory disease (APR = 1.34; 95% CI, 1.23–1.46); lack of reliable transportation was the strongest measure associated with stroke (APR = 1.34; 95% CI, 1.18–1.53); life dissatisfaction was the strongest measure associated with diabetes (APR = 1.15; 95% CI, 1.05–1.25); mental stress was the strongest measure associated with current asthma (APR = 1.35; 95% CI, 1.26–1.44), arthritis (APR = 1.33; 95% CI, 1.29–1.38), cancer (APR = 1.27; 95% CI, 1.17–1.38), and depression (APR = 1.83; 95% CI, 1.77–1.90). Social isolation/loneliness was also strongly associated with depression (APR = 1.82; 95% CI, 1.76–1.89).

**Figure:**
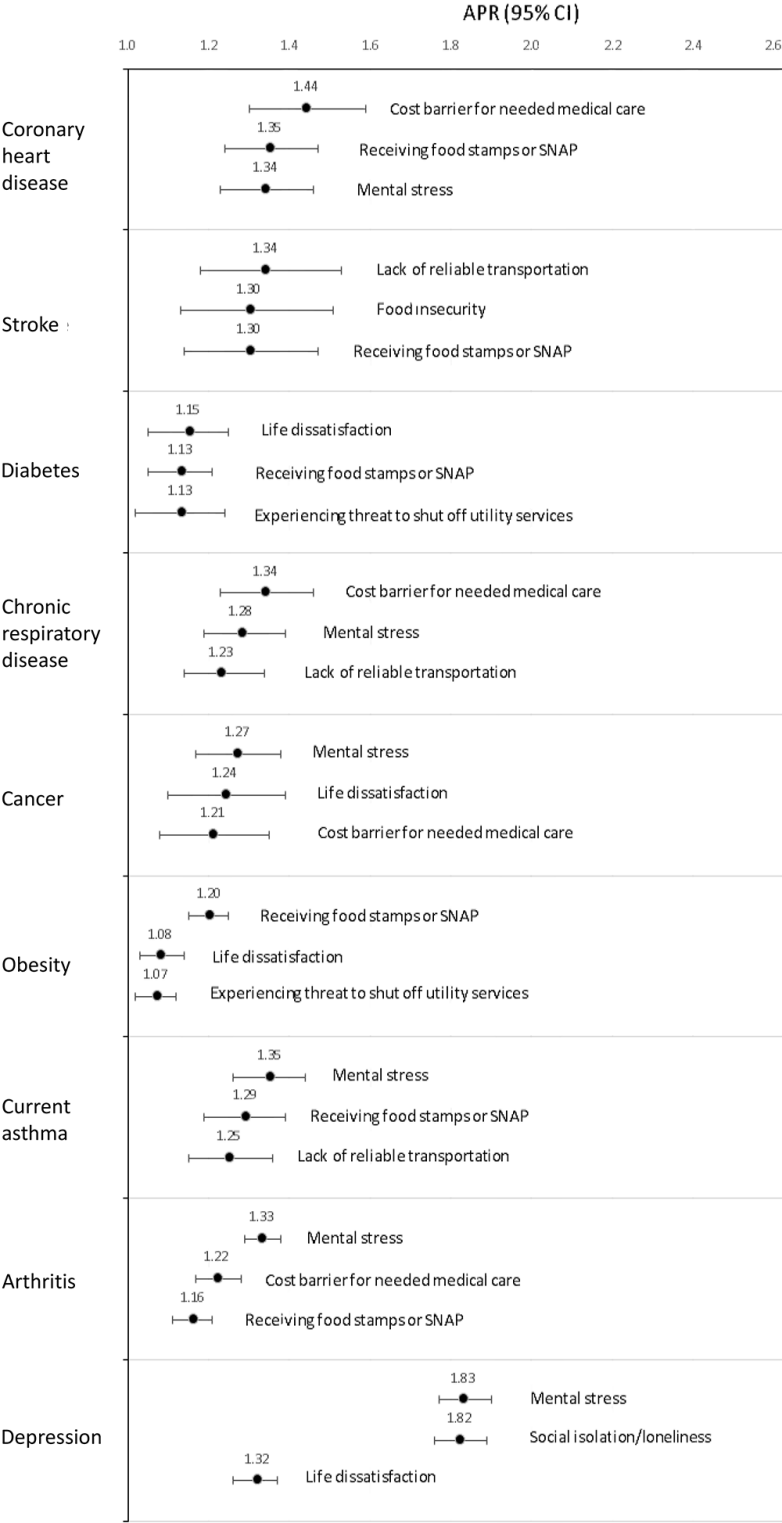
Leading SDOH/HRSN measures associated with 9 chronic diseases among US adults in 39 states, the District of Columbia, and 2 US territories, Behavioral Risk Factor Surveillance System, 2022. Adjusted prevalence ratios (APRs) were adjusted for demographic variables and disease-specific risk factors, ie, current smoking, physical activity, alcohol use, obesity, and diabetes for coronary heart disease, and stroke; current smoking, physical activity, alcohol use, and obesity for diabetes, chronic respiratory disease, cancer, current asthma, arthritis, and depression; and current smoking, physical inactivity, and alcohol use for obesity. Abbreviations: CHD, coronary heart disease; SDOH/HRSN, social determinants of health/health-related social needs; SNAP, Supplemental Nutrition Assistance Program.

**Table:** 

Chronic disease and SDOH/HRSN measure	APR (95% CI)
**CHD**	
Cost barrier for needed medical care	1.44 (1.30–1.59)
Receiving food stamps or SNAP	1.35 (1.24–1.47)
Mental Stress	1.34 (1.23–1.46)
**Stroke**	
Lack of reliable transportation	1.34 (1.18–1.53)
Food insecurity	1.30 (1.13–1.51)
Receiving food stamps or SNAP	1.30 (1.14–1.47)
**Diabetes**	
Life dissatisfaction	1.15 (1.05–1.25)
Receiving food stamps or SNAP or food insecurity	1.13 (1.05–1.21)
Experiencing threat to shut off utility services	1.13 (1.02–1.24)
**Chronic respiratory disease**	
Cost barrier for needed medical care	1.34 (1.23–1.46)
Mental stress	1.28 (1.19–1.39)
Lack of reliable transportation	1.23 (1.14–1.34)
**Cancer**	
Mental stress	1.27 (1.17–1.38)
Life dissatisfaction	1.24 (1.10–1.39)
Cost barrier for needed medical care	1.21 (1.08–1.35)
**Obesity**	
Receiving food stamps or SNAP	1.20 (1.15–1.25)
Life dissatisfaction	1.08 (1.03–1.14)
Experiencing threat to shut off utility services	1.07 (1.02–1.12)
**Current asthma**	
Mental stress	1.35 (1.26–1.44)
Receiving food stamps or SNAP	1.29 (1.19–1.39)
Lack of reliable transportation	1.25 (1.15–1.36)
**Arthritis**	
Mental stress	1.33 (1.29–1.38)
Cost barrier for needed medical care	1.22 (1.17–1.28)
Receiving food stamps or SNAP	1.16 (1.11–1.21)
**History of depression**	
Mental stress	1.83 (1.77–1.90)
Social isolation/loneliness	1.82 (1.76–1.89)
Life dissatisfaction	1.32 (1.26–1.37)

## Discussion

For the first time, the new SD/HE BRFSS module provides an opportunity to analyze individual reports of HRSN and established population-based SDOH unique to specific chronic diseases among the US adult population. Although this study is correlative and does not establish causality (having multiple chronic diseases could either lead to economic and social risks or be caused by them), information on the relationship between chronic diseases and SDOH/HRSN is critical for the health care delivery system and the public health system. Chronic conditions continue to be among the leading causes of death and health care cost in the US (2). As the population ages, the burden will increase. Additional studies that stratify data by demographic characteristics, including age, would be of interest.

This study found that, in general, people with chronic conditions were more likely than those without chronic conditions to have SDOH/HRSN. And having more than 1 chronic disease was associated with having more SDOH/HRSN. These findings are similar to those identified in studies that used data from the electronic health records of selected health care systems (17,29). Examining these findings with self-reported data on a national scale can help guide public health policy and practice. One notable exception identified in this study was that survey participants with cancer did not have more SDOH/HRSN than those without cancer. This finding was puzzling considering that people with cancer are known to have many HRSN (30). In this study, we do not know whether those answering affirmatively to the cancer question were currently in treatment or were long-term survivors. As noted previously, circumstances of survivors differ extensively (31). To date, we have not identified any research that compared HRSN among cancer patients and survivors with HRSN among people with other chronic diseases. We do know that patient navigation programs support cancer patients through their cancer journey (emotional support, social service connection, care coordination) (32), but such programs are not as common for other chronic conditions. This additional support may be a possible explanation for our findings. In addition, cancer survivors have many different paths and their needs are highly dependent on factors such as age, sex/gender, financial and job status, race and ethnicity, and sequelae from treatment (30). Without this information, it is difficult to understand our findings. More research on the HRSN of cancer patients and survivors is merited.

The leading SDOH/HRSN associated with chronic diseases differed somewhat by condition, but overall they were similar to those identified in prior research: food security, transportation, and cost of medical care (33). Given that most of our study participants (91.2%) had health insurance, we speculate that cost of medical care was a concern because of out-of-pocket deductibles, co-pays, or co-insurance. As Medicare and statewide Medicaid programs begin reimbursing for social need interventions, it will be important to understand how these HRSN affect other health care costs. The concern about cost of medical care may also be due to high levels of health care use associated with chronic conditions and was particularly important for people with depression. A recent Kaiser report noted that patients with depression and/or anxiety who had private insurance had almost twice the annual out-of-pocket spending as patients without these diagnoses. Some of this spending may be due to increased use of health care services, but some may be due to difficulties with in-network access (34). In addition to aforementioned SDOH/HRSN, mental stress was a major issue for participants with chronic diseases and indicates the challenges inherent in having 1 or more chronic diseases.

Of interest is that some chronic diseases are more or less related to specific HRSN. For example, depression is highly correlated with loneliness and social isolation. The relationship found between stroke and lack of transportation could be explained by challenges posed by poststroke disability. Driving capacity may be limited by the need for specialized transportation. The differences in specific chronic diseases and reported HRSN were noted in a study by Heller et al (17).

Fulfilling individual HRSN may help to improve health care outcomes and costs (16). However, even as the health care system begins screening and referring for HRSN, access to resources varies in any given community. This community-level variability highlights the need for community-level action to address SDOH. The work of multiple-sector partnerships to implement policy, systems, and environmental changes to improve community health is a critical shared responsibility (35). Having data on populations most vulnerable to adverse SDOH/HRSN at the state and community level can help planners target resources effectively.

CDC recently released small-area estimates on 7 items from the BRFSS SD/HE module in PLACES (36,37). For the District of Columbia and the 39 states that fielded the module, hyperlocal data on the prevalence of SDOH/HRSNs are now publicly available at all levels of geography (ie, county, city, town, and neighborhood). Future studies can examine the relationships among SDOH/HRSN and chronic diseases stratified by geographic region. Understanding how specific SDOH/HRSN relate to specific chronic conditions will assist local public health decision makers with program planning and resource allocation to improve population health. Similarly, health care providers can use the data to geographically identify and address the health needs of their patients, and social services providers can use the data to assess where services are needed most. Collectively, health care, public health, and human services can work together to identify, prioritize, and improve the health and well-being of the populations they serve.

### Limitations

The study has several limitations. First, the BRFSS is a national telephone-based survey that collects data from a representative sample generated by each state. As a result, response rates vary by state, which may not be ideal. However, the application of sampling weights likely reduces the effect of some nonresponse bias. Also, data are based on self-report and presence or absence of a chronic condition, and these data are not validated with medical information. Institutionalized populations are not included. Data for the SD/HE module were collected for 39 states, the District of Columbia, and 2 US territories, which limits the generalizability of the results to the US population. Sample sizes varied based on the nonmissing SDOH/HRSN responses. Finally, the questions on the SD/HE module represent a subset of SDOH/HRSN that can affect people’s daily lives; they do not address determinants such as discrimination or neighborhood safety.

### Conclusions

People with chronic disease conditions are likely to have several SDOH/HRSN. From a treatment and prevention perspective, providers should consider the influence of SDOH/HRSN on those with or at risk for chronic diseases. In communities with high rates of chronic disease, focusing on correlated SDOH for collective action may be most beneficial. Given that individuals with chronic conditions may be especially vulnerable to adverse SDOH/HRSN during emergencies, population-based information on SDOH can support community resilience priorities and underpin plans for mitigation.
